# Mortality and cardiac arrest rates of emergency surgery in developed and developing countries: a systematic review and meta-analysis

**DOI:** 10.1186/s12871-024-02559-w

**Published:** 2024-05-20

**Authors:** Kaikai Guo, Fengying Xu, Ye Li, Mingxing Ma, Jing Li, Long Wang

**Affiliations:** 1 Department of pain medicine, The First Medical Center, Chinese PLA General Hospital, 28 Fuxing Road, Beingjing, China; 2 Department of anesthesiology, No. 971 Hospital of People’s Liberation Army Navy, Qingdao, China; 3 Department of acupuncture, The First Medical Center, Chinese PLA General Hospital, Beijing, China

**Keywords:** Perioperative mortality, Cardiac arrests, 30-day postoperative mortality, Emergency surgery

## Abstract

**Background:**

The magnitude of the risk of death and cardiac arrest associated with emergency surgery and anesthesia is not well understood. Our aim was to assess whether the risk of perioperative and anesthesia-related death and cardiac arrest has decreased over the years, and whether the rates of decrease are consistent between developed and developing countries.

**Methods:**

A systematic review was performed using electronic databases to identify studies in which patients underwent emergency surgery with rates of perioperative mortality, 30-day postoperative mortality, or perioperative cardiac arrest. Meta-regression and proportional meta-analysis with 95% confidence intervals (CIs) were performed to evaluate global data on the above three indicators over time and according to country Human Development Index (HDI), and to compare these results according to country HDI status (low vs. high HDI) and time period (pre-2000s vs. post-2000s).

**Results:**

35 studies met the inclusion criteria, representing more than 3.09 million anesthetic administrations to patients undergoing anesthesia for emergency surgery. Meta-regression showed a significant association between the risk of perioperative mortality and time (slope: -0.0421, 95%CI: from − 0.0685 to -0.0157; *P* = 0.0018). Perioperative mortality decreased over time from 227 per 10,000 (95% CI 134–380) before the 2000s to 46 (16–132) in the 2000–2020 s (*p* < 0–0001), but not with increasing HDI. 30-day postoperative mortality did not change significantly (346 [95% CI: 303–395] before the 2000s to 292 [95% CI: 201–423] in the 2000s-2020 period, *P* = 0.36) and did not decrease with increasing HDI status. Perioperative cardiac arrest rates decreased over time, from 113 per 10,000 (95% CI: 31–409) before the 2000s to 31 (14–70) in the 2000–2020 s, and also with increasing HDI (68 [95% CI: 29–160] in the low-HDI group to 21 [95% CI: 6–76] in the high-HDI group, *P* = 0.012).

**Conclusions:**

Despite increasing baseline patient risk, perioperative mortality has decreased significantly over the past decades, but 30-day postoperative mortality has not. A global priority should be to increase long-term survival in both developed and developing countries and to reduce overall perioperative cardiac arrest through evidence-based best practice in developing countries.

**Supplementary Information:**

The online version contains supplementary material available at 10.1186/s12871-024-02559-w.

## Introduction

The risk of death associated with surgery and anaesthesia can vary considerably. Typically, mortality is lower in the perioperative period than in other periods [1]. The rate of cardiac arrest can be twice as high in patients receiving anesthesia for emergency surgery compared with elective surgery. In addition, mortality is much higher in the former population [2–6]. Despite this, accurate quantification of perioperative mortality and cardiac arrest rates remains a challenge.

The Safe Surgery Checklist has been published to reduce morbidity and mortality associated with surgery in both developed and developing countries [7]. However, emergency surgery is an unplanned procedure with rushed anesthesia, more blood loss and injury, leading to increased surgery-related morbidity and mortality compared to elective surgery. In an emergency, patients often lack adequate preoperative care [8]. Although current surgery and general anesthesia are safer and more sophisticated than in the past [9], a systematic analysis of mortality associated with emergency surgery has not been performed.

Mortality and safety associated with emergency surgery may vary between regions with different medical resources [10]. In this study, we conducted a meta-analysis of research data on anesthesia- and surgery-related deaths in emergency surgery in developed and developing regions to examine whether this mortality has decreased over time.

Several studies have shown that mortality varies over different time periods, such as intraoperative, in-hospital and 30-day mortality [11, 12]. However, Rodney et al. reported that admission time was not associated with increased mortality in emergency surgery, which contradicts the findings of other studies. Therefore, we performed a subgroup analysis in our meta-analysis to further investigate this discrepancy.

## Methods

We searched for studies reporting perioperative and/or anesthesia-related cardiac arrest and mortality in patients receiving emergency surgery. The follow-up period could extend up to the 30th postoperative day. Relevant studies were retrieved out from the PubMed, EMBASE, LILACS, and SciELO databases from inception to December 30, 2022. Important publications traced back earlier were also screened. Index terms (e.g., MeSH and Emtree), text terms, as well as words having the same meaning as “an(a)esthesia”, “cardiac arrest”, “mortality” and “emergency or urgency surgery” were entered. The studies were searched according to the strategy for each database. Titles and abstracts were screened, and full texts of potentially eligible studies were independently reviewed by two investigators (GKK and LY). Three authors (WL, XFY and LJ) independently checked the references of all systematic reviews, observational studies, and treatment guidelines to identify relevant literature. Any missing information was collected from the authors of publications through communication. Studies reported in any language were acceptable, and could be translated to facilitate screening.

To ensure statistical significance, studies involving anesthesia for emergency surgery in hospitals should have a study population of at least 1000 patients, as the incidence of death or cardiac arrest is typically less than 1/1000. For the same reason, we excluded studies that report regional or local anesthesia performed outside of hospitals. We also excluded studies that focused solely on one population subtype (e.g., only the elderly or only children) or one specific type of surgery (e.g., only cardiac surgery). Finally, we excluded studies that reported only estimated denominator or only a portion of adverse events from a larger population, rather than an actual incidence, or that only reported mortalities corrected with morbidity scoring systems without raw data.

The primary outcome was perioperative anesthesia or surgery related mortality, and the secondary outcome was the mortality within 30 days after surgery and the rate of anesthesia-related cardiac arrest.

### Study quality assessment and data extraction

Two independent reviewers (GKK and MMX) undertook study quality assessment and data extraction, and any discrepancies were resolved with input from the third reviewer (WL). For studies of rates of anesthesia or surgery related death, we assessed the following criteria: representativeness of the population, sample selection, prospective or retrospective, adequacy of sample size, and outcome assessment. We deemed a study to be adequate for representativeness if it included institutions from various settings such as rural and urban hospitals in a region or country, and to be inadequate if it included only one hospital or unit. We classed sample selection as adequate if all patient deaths of emergency operation were included, and as inadequate if a particular group of patients were excluded. We deemed outcome assessment to be adequate when a confidential inquiry, verbal autopsy, or professional panel established the cause of death and inadequate when there was no special eff ort or use of registry data from only one source. An adequate sample size included data for at least 3000 operations. A study was classed as high quality if three of the above five criteria were met.

To classify countries based on their human development index (HDI) (Retrieved from online data at OurWorldInData.org), a cut-off of ≥ 0.800 was set for high-HDI countries, and < 0.800 for low-HDI countries. Since the HDI of a country can change over time, and studies often report data for a period, the HDI of each country was calculated as the HDI value in the median year of the study period. If the HDI value for a particular year was not available, it was imputed using the HDI value from the previous, next or nearest year.

### Statistical analysis

The rate of each event was calculated as the number of occurrences per 10,000 anesthetic procedures, along with their corresponding 95% confidence intervals (CIs). To account for heterogeneity, random effects model was used to calculate weighted proportions across all studies using a generalized linear mixed-effects model. The time and HDI were dichotomized into pre-2000 versus 2000-present and low HDI versus high HDI, respectively. As many studies reported data over multiple years, we collected data from the most recent year of the recruitment period. If data were only provided in aggregate time intervals, we used the median year to represent this recruitment period.

Mortality rates reported in studies vary widely due to many influencing factors, which can greatly affect the accuracy of the regression. Therefore, we used the Dixon’s Q test to identify and remove outliers before performing meta-regression (Dixon, W. J.). We then performed a meta-regression with random-effects model with an observed log-odds ratio to predict whether anesthesia-related mortality and cardiac arrest rates changed significantly by time or country’s HDI status (time and HDI as continuous variables).

We used I^2^ to quantifies the effect of heterogeneity, which indicates the proportion of variability between studies resulting from heterogeneity rather than sampling errors [13]. An I^2^ value greater than 50% suggests significant heterogeneity among studies. Statistical significance was considered as a two-sided *P*-value of less than 0.05. Statistical analysis was performed using the R software (The R Foundation for Statistical Computing, version 4.1.2) with packages “meta”, “outliers” and “metafor”.

## Results

**Fig. 1:**
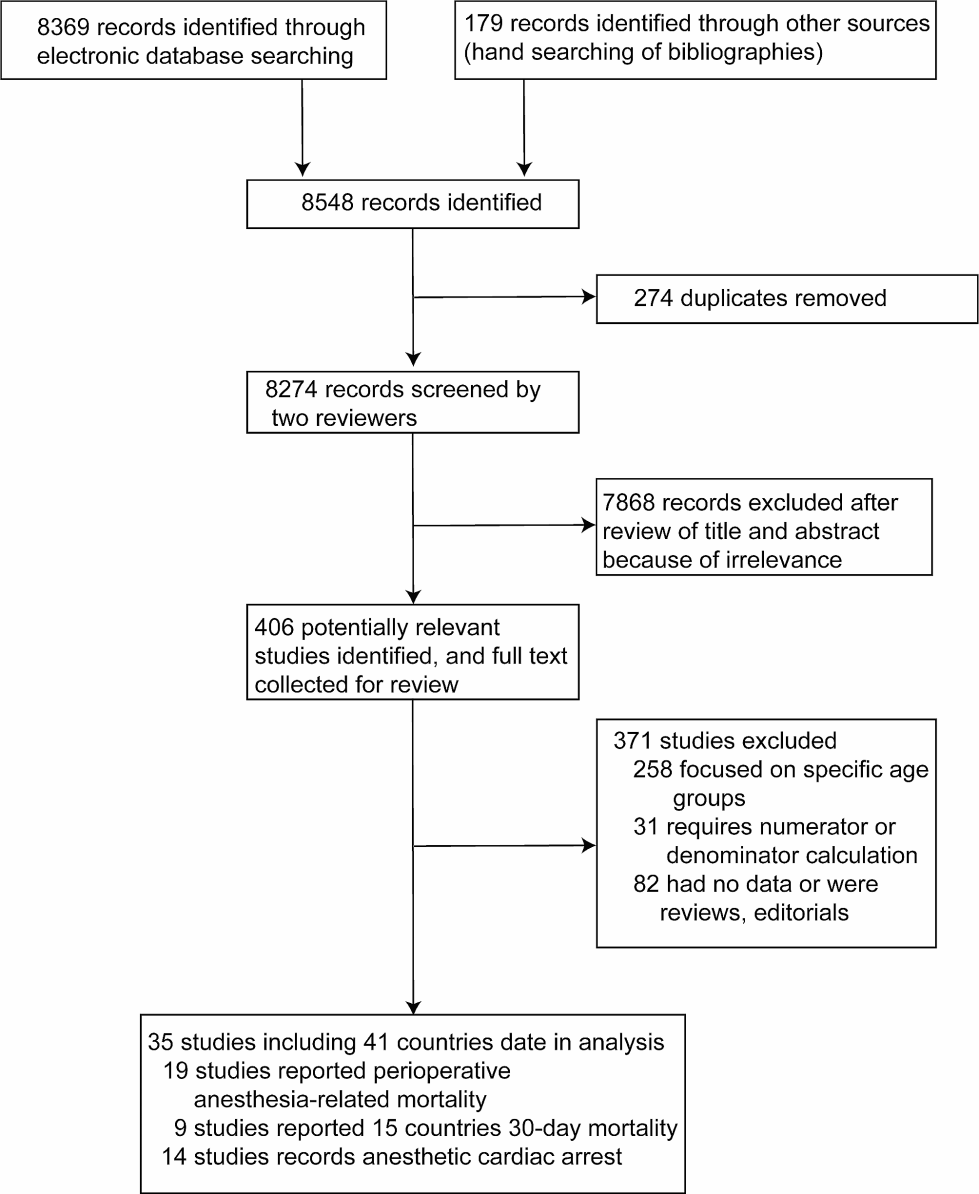
Flowchart of study identification

A total of 8548 abstracts and 406 potentially relevant full-text articles were screened (refer to Fig. 1). Out of these, 35 studies covering 41 countries were found to be eligible, involving more than 3.09 million anesthetic administrations for emergency surgery (see Appendix Table 1 for study characteristics and designs). Nineteen studies reported perioperative anesthesia-related mortality, which mainly occurred intraoperatively or within the first 24–48 h postoperatively. Four studies reported post-anesthesia 9-day following anesthesia, while fifteen studies reported 30-day mortality after emergency surgery. Anesthetic cardiac arrest was reported in 14 of the included studies.

**Table 1:** Anesthesia mortality and cardiac arrests by decade and by country human development index status

	Events	Weighted event rate per 10,000 (95%CI)	*p* value for subgroup interaction
**Perioperative mortality**			
*by decade*			
pre-2000s	1,967/61,483	227 (134–380)	*P* < 0.0001*
2000s-now	641/139,384	46 (16–132)	
*by HDI*			
Low-HDI	424/40,456	126 (42–366)	*P* = 0.87
High-HDI	2,184/160,411	138 (51–364)	
**30-day mortality**			
*by decade*			
pre-2000s	209/6,044	346 (303–395)	*P* = 0.36
2000s-now	84,102/2,545,384	292 (201–423)	
*by HDI*			
Low-HDI	804/21,984	345 (171–686)	*P* = 0.44
High-HDI	85,309/2,529,444	267 (171–413)	
**Cardiac arrests**			
*by decade*			
pre-2000s	287/29,676	113 (31–409)	*P* = 0.015*
2000s-now	1,072/158,826	31 (14–70)	
*by HDI*			
Low-HDI	1,159/116,353	68 (29–160)	*p* = 0.012*
High-HDI	200/72,149	21 (6–76)	

^*^a statistically significant difference between the two groups. HDI: human development index status; High-income countries were defined as HDI > 0.8 and low-income countries as HDI < 0.8.

**Fig. 2:**
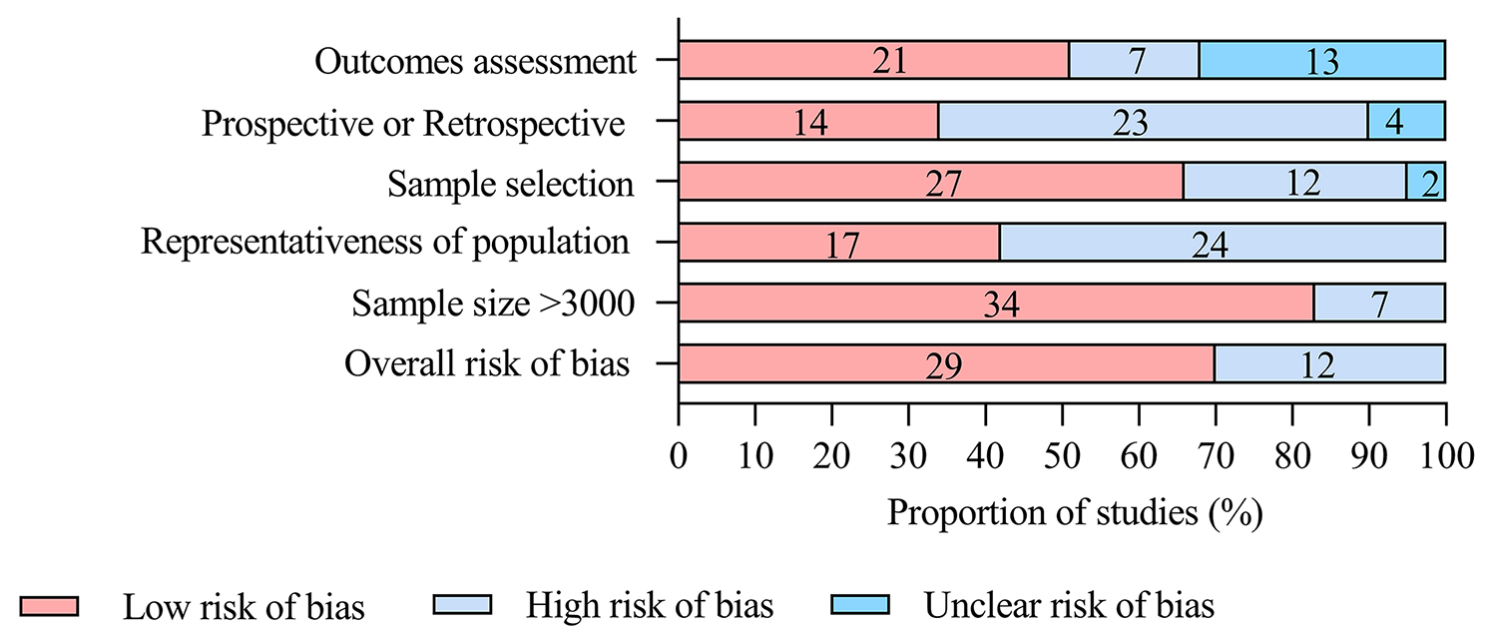
Risk of bias assessment

Out of the 41 countries analyzed for anesthesia-related mortality, 29 (70.7%) had a low risk of bias, 24 (58.5%) had a high representativeness of the population and setting, 27 (65.9%) had adequate sample selection, and 20 (48.7%) had appropriate reporting of outcomes. Additionally, 34 (82.9%) studies had an adequate sample size, and 23 (56.1%) were retrospective (Fig. 2). Statistical heterogeneity (I^2^ > 50%) was detected in all event rates.

### Meta-regression analysis (Figs. 3 and 4)

**Fig. 3:**
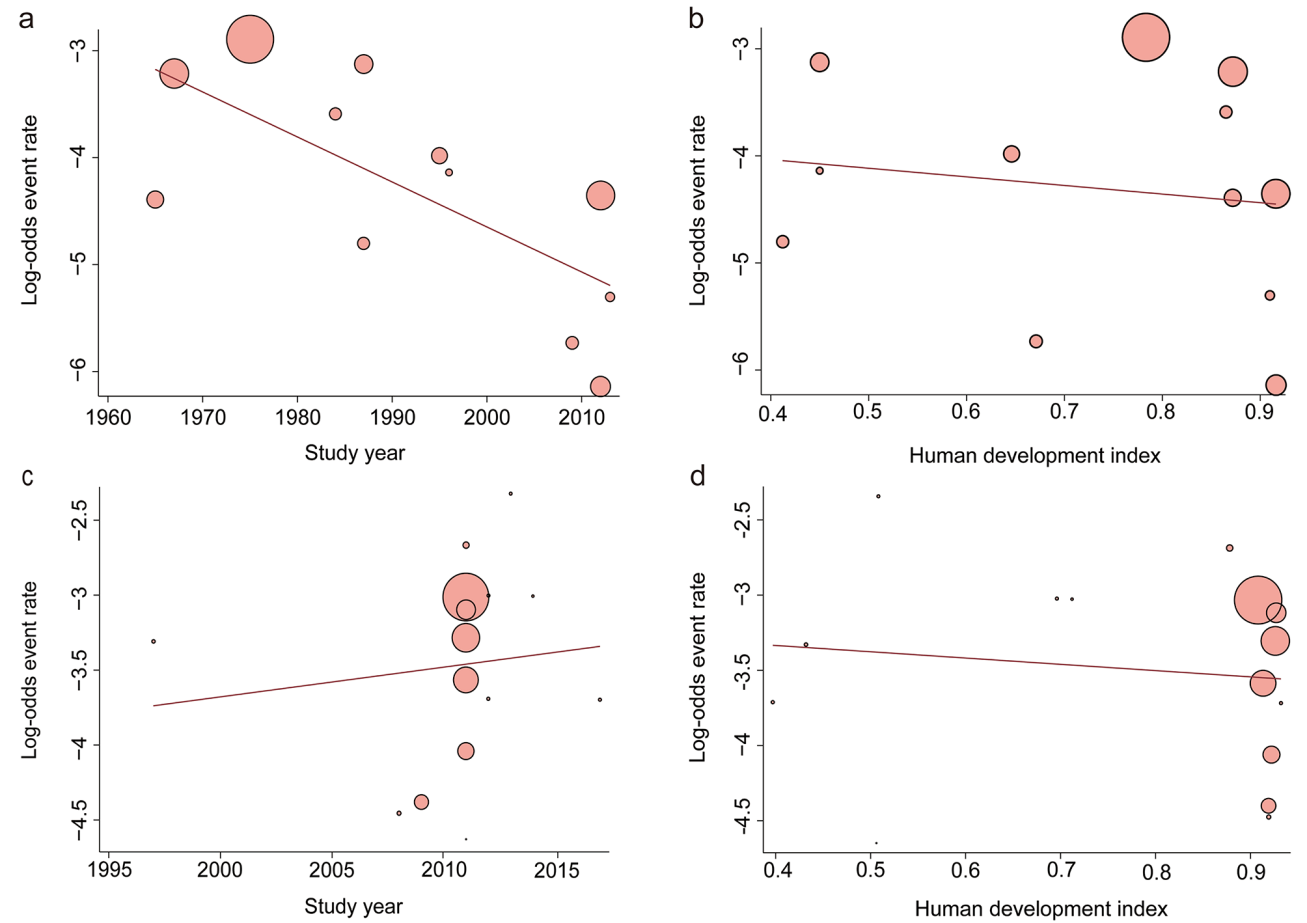
Meta-regression of perioperative mortality (**a, b**), 30-day mortality (**c, d**) outcomes by decades (**a, c**) and country’s Human Development Index status (**b, d**). Every circle represents a study; the circle size is representative of the weight of that study in the analysis

**Fig. 4:**
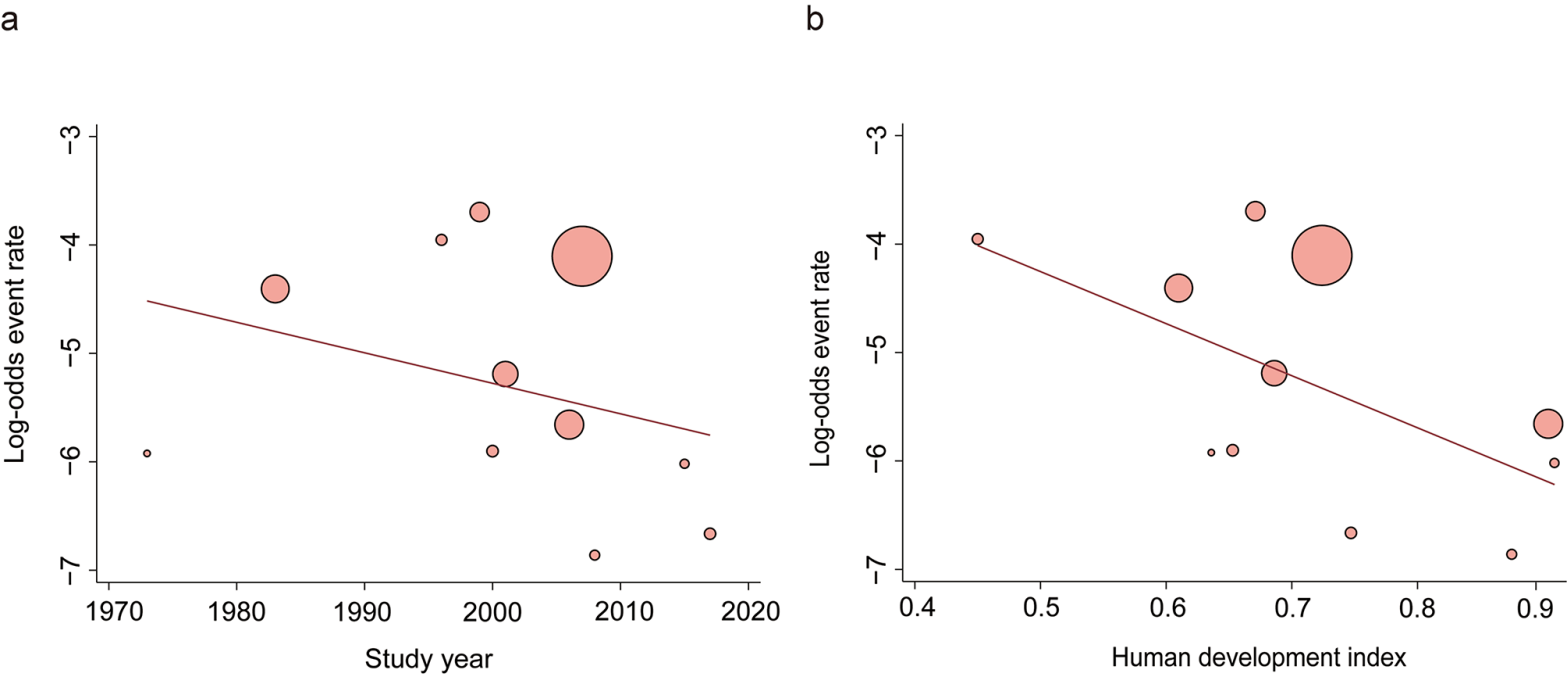
Meta-regression of perioperative cardiac arrest (a, b) rates outcomes by decades (**a**) and country’s Human Development Index status (**b**). Every circle represents a study; the circle size is representative of the weight of that study in the analysis

#### Perioperative mortality

The perioperative mortality reported in seven studies (Richard 1985; Pedersen 1989; Biboulet 1998; Choi 2014; Kim 2020; Karalliedde 1975; Ahmed 2008, see in appendix Table 1) was identified as outliers by the Dixon’s test. The largest value among these studies was 0.2%, which is smaller than the mean value of 1.9% in the remaining studies. After removing the outliers, meta-regression revealed a significant decrease in perioperative mortality over time (slope: -0.0421; 95% CI: -0.0685 to -0.0157; *P* = 0.0018). However, there was no significant correlation between perioperative mortality and HDI (slope: -0.8032; 95% CI: -4.0079 to 2.4015; *P* = 0.6233).

#### 30-day mortality

Dixon’s test did not identify any outliers. Meta-regression analysis showed that there was no significant relationship between time and 30-day mortality (slope: 0.0196, 95% CI: -0.0653 to 0.1044; *P* = 0.6510). Similarly, there was no significant decrease in 30-day mortality with increasing HDI (slope: -0.3539, 95% CI: -2.1237 to 1.4160; *P* = 0.6952).

#### Perioperative cardiac arrest rates

The rates reported in three studies (Richard 1985; Biboulet 1998; Ahmed 2008, see in appendix Table 1) were identified as outliers by the Dixon’s test. The largest value among these studies was 0.06%, which is smaller than the mean value of 0.8% in the remaining studies. After removing the outliers, meta-regression revealed a significant the relationship between perioperative cardiac arrest rates and time was significant (slope: -0.0421, 95% CI: -0.0721 to -0.0120; *P* = 0.0108). However, there was a significant decrease in the relationship between perioperative cardiac arrest rates significantly decreased and increasing HDI (slope: -4.7763; 95% CI: -8.9403 to -0.6122; *P* = 0.0246).

### Proportional meta-analysis (Table 1)

The meta-analysis found a significant difference in perioperative mortality rates before and after 2000 (227 per 10,000; 95% CI, 134–380 versus 46 per 10,000; 95% CI, 16–132, *P* < 0.001). However, there was no significant difference in rates between low-HDI and high-HDI countries (126 per 10,000; 95% CI, 42–366 vs. 138 per 10,000; 95% CI, 51–364, *P* = 0.87).

The included studies had an average 30-day mortality rate of 3.5%. There was no significant difference in the 30-day mortality rate after emergency surgery between the pre-2000 and post-2000 groups (346 per 10,000; 95%CI, 303–395 versus 292 per 10,000; 95%CI, 201–423, *P* = 0.36), nor. between low-HDI and high-HDI countries (345 per 10,000; 95% CI: 171–686 versus 267 per 10,000; 95% CI: 171–413, *P* = 0.44).

The occurrence of perioperative cardiac arrest was significantly lower in the post-2000 group (31 per 10,000; 95% CI, 14–70) compared to the pre-2000 group (113 per 10,000; 95% CI, 31–409, *P* = 0.015). Additionally, the incidence of perioperative cardiac arrest was much lower in high-HDI countries (21; 95% CI, 6–76) than in low-HDI countries (68; 95% CI, 29–160, *P* = 0.012).

## Discussion

In the last ten years, researchers have extensively analysed the risk factors for mortality and cardiac arrest in patients receiving anaesthesia for surgery [2, 4–6]. However, most studies have focused on patients undergoing elective surgery, with only occasional attention given to emergency surgery [2, 4–6, 14]. Anesthesia is typically well-planned for elective surgery, but hastily administered before emergency surgery, which increases the risk of associated mortality. This study employed meta-regression and weighted meta-analysis to show that perioperative mortality has significantly decreased in recent decades (pre-2000 vs. after-2000). However, 30-day mortality has remained unchanged over time. There were no significant differences in both overall perioperative mortality and 30-day mortality between high-HDI and low-HDI countries. Furthermore, perioperative cardiac arrest rates decreased significantly with an increase in HDI, but not with time.

Safety in anesthesia has been a major concern since the early 1980s. The present study shows a steady decrease in anesthesia-related and perioperative mortalities. Advancements in anesthesia and surgery, including improved asepsis, drugs and techniques, better control of contraindications antibiotic medication, physiological monitoring, fluid and blood management, post-operative critical care, and perioperative health education, may have contributed to this. However, the 30-day mortality rate after emergency surgery has not decreased over time and remains unchanged between developing and developed countries.

A systematic review and meta-analysis of 87 studies conducted across 25 countries from 1944 to 2009 revealed that patients underwent a total of 21.4 million anesthetic operations for surgery. The study found a decrease in mortality related to both entirely and partially administered anesthesia over the decades [9], which is consistent with the change in perioperative mortality related to emergency surgery found in the present study. However, we observed that the 30-day mortality rate did not decrease over time. Another possible reason is that, despite advancements in medical care and anesthetic management, long-term mortality rates have not improved significantly. It is important to conduct further research to fully understand the factors contributing to this trend. One reason for this lack of significant decline in long-term mortality could be due to the fact that studies conducted before 2010 had smaller sample sizes and only provided data on 30-day mortality. Thus, the 30-day mortality rate prior to 2010 was 346 per 10,000 (95% CI 303–395), which may have decreased in the following decade. The 30-day postoperative mortality and inpatient mortality are both commonly used indicators for post-surgery outcomes. The 30-day postoperative mortality and inpatient mortality are both commonly used indicators for post-surgery outcomes. While the former is a strong worldwide indicator, the latter is easier to obtain. The 30-day postoperative mortality and inpatient mortality are both commonly used indicators for post-surgery outcomes. Obtaining the 30-day mortality requires close follow-up for 30 days after emergency surgery. Therefore, the Lancet Commission on Global Surgery recommends perioperative inpatient mortality as a key indicator for surgical outcomes [15]. However, modern communication tools allow for close monitoring even in underdeveloped regions, which enhances the potential applicability of postoperative 30-day mortality in clinical practice [16]. Studies published between 2011 and 2020 have shown lower anesthesia-related mortalities in countries with very high HDI, ranging from 0 to 215.6 per million anesthesia cases [17–20], compared to high-HDI countries, which had rates ranging from 0 to 951.4 per million cases [21, 22]. During the intraoperative to postoperative three-month period, countries with a very high HDI had a low anesthesia-related mortality rate of 4.19 per million anesthesia cases [23]. From postoperative 24 h to 30 days, the mortality rate was 0 per million anesthesia cases [24]. This meta-analysis found that mortality during the perioperative period and the 30 days after emergency surgery did not significantly decrease in high-HDI countries compared to low-HDI countries. This could be explained by the tendency of higher-HDI countries to report outcomes of older or more severe patients.

A meta-regression analysis including a total of 1,975,964 anesthetics administered to patients undergoing all types of surgery, did not find a significant relationship between the global perioperative cardiac arrest rate and time [25]. Our review, which included 188,502 patients undergoing emergency surgery showed a significant decline in perioperative cardiac arrest rate. The lower perioperative cardiac arrest rates in high-income countries demonstrate significant improvement in patient anesthesia safety in these developed nations. Despite the fact that pulse oximetry and capnography were first developed in the early 1970s, they were not routinely used until a decade later in high-HDI countries [26]. Unfortunately, even in the 2000s, the routine use of pulse oximetry and capnography is not universal in some low-income countries [27, 28]. For high-risk patients, continued monitoring in an intensive care unit may reduce anesthesia-related morbidity and mortality; Failure to provide or use these facilities may increase perioperative cardiac arrest rates and mortality [29].

It is important to acknowledge that the universality of the findings is limited. This is due to differences in the included studies, such as study populations (e.g. exclusion of pediatric patients or patients in ASA V physical status in some studies), study period (i.e. intraoperative, postoperative 24 h, or postoperative 7 days), and surgery modes (e.g. exclusion of cardiac, trauma, or obstetrical surgeries in some studies). These differences could have resulted in substantial heterogeneity. A random-effects model for meta-analysis was established as a result. Data were collected from single or multiple centers, or even a national cohort. To reduce bias, only large studies (> 1000 patients) were included in the present study, with weighted event rates calculated across all these studies. Additionally, many of the included studies are limited by underreporting of perioperative and anesthesia-related cases. Publication bias is a concern as some studies reporting high mortality may not have been published due to institutions or surgeons not wanting to damage their reputation. Similarly, studies reporting low mortality may also have gone unpublished. Additionally, our data may primarily reflect mortality rates at high-level medical centers rather than those with limited medical resources. The definition of anesthesia-related cardiac arrest and death is a topic of controversy and requires a consensus [30, 31]. In conclusion, the perioperative and 30-day postoperative mortalities are comparable between developing and developed countries. The perioperative mortality related to emergency surgery has steadily declined over the past 50 years. However, the 30-day mortality after emergency surgery remains unchanged over time, regardless of HDI status. Perioperative cardiac arrest rates decrease with time and also with increasing HDI. Efforts should be made globally to decrease the 30-day postoperative mortality rate in both developed and developing countries.

### Electronic supplementary material

Below is the link to the electronic supplementary material.

Supplementary Material 1

Supplementary Material 2

Supplementary Material 3

## Data Availability

This datasets used and/or analyzed during the current study are available from the corresponding author an reasonable request.

## Abbreviations

CIs: Confidence intervals
HDI: Human development index
