# Supplementary material for: Mortality and cardiac arrest rates of emergency surgery in developed and developing countries: a systematic review and meta-analysis
Source: BMC Anesthesiol. 2024 May 20;24:178. doi: 10.1186/s12871-024-02559-w (PMC11104000; doi:10.1186/s12871-024-02559-w)
Supplement: Supplementary file 1 — Supplementary Material 1 [file 12871_2024_2559_MOESM1_ESM.docx]

**Table 1: Appendix Table 1 Description of the included studies of operation-related mortality and cardiac arrest rates outcomes**

| **Investigator and year of publication** | **Data source** | **Country** | **Study period** | **Median**  **year** | **Cardiac**  **arrest (n)** | **Death**  **(n)** | **Patients**  **(n)** | **Time period of mortality** | **Cardiac arrest rate*** | **Mortality rate*** | **Survival**  **n (%)** | **Excluded** |
| --- | --- | --- | --- | --- | --- | --- | --- | --- | --- | --- | --- | --- |
| **Cardiac arrest** | | | | | | | | | | | | |
| L. D. Karalliedde et al.1975 | General Hospital | Sri Lanka | 1972-1973 | 1973 | 8 |  | 2995 | Perioperative | 26.71 |  |  |  |
| Richard L. Keenan et al.  1985 | Medical College of Virginia | USA | 1969-1983 | 1976 | 10 |  | 15300 | Within 9 days of operation | 6.66 |  |  |  |
| P. P. Ruiz Neto et al.  1986 | University of Sao Paulo School of Medicine | Brazil | 1982-1984 | 1983 | 154 |  | 12770 | Perioperative | 120.60 |  |  |  |
| Philippe Biboulet et al. 1998 | Lapeyronie University hospital | France | 1989-1995 | 1992 | 2 |  | 21676 | The first 12 postoperative  hours in the PACU or ICU | 0.92 |  |  | ASA 5 |
| A.A. Sanusi et al.  2001 | University College Hospital | Nigeria | 1994-1998 | 1996 | 24 |  | 1274 | Operating room | 188.38 |  |  |  |
| Leandro Gobbo Braz et al. 2004 | Hospital das Clinicals | Brazil | 1996-2002 | 1999 | 75 |  | 3099 | Perioperative | 242.01 |  |  |  |
| A. Ahmed et al.  2008 | Aga Khan University Hospital | Pakistan | 1992-2006 | 1999 | 16 |  | 24700 | Hospital discharge | 6.48 |  |  | Cardiac surgery |
| Naiyana- Aroonpruksakul et al. 2002 | Siriraj Hospital | Thailand | 1999-2001 | 2000 | 26 |  | 9538 | Within 48 hours of operation | 27.26 |  |  |  |
| L. G. Braz et al.  2006 | Singlg Tertirary Teaching Hospital | Brazil | 1996-2005 | 2000 | 126 |  | 22747 | Operation room and PACU | 55.39 |  |  |  |
| Sumeet Goswami et al. 2012 | American College of Surgeons National Surgical Quality Improvement Program | USA | 2005-2007 | 2006 | 165 |  | 47415 | Intraoperative | 34.80 |  |  | Cardiac surgery, monitored anesthesia care, local anesthesia, trauma, transplant and <16yr of age, brain-dead patients |
| Visith siriphuwanun  et al. 2014 | Maharaj Nakorn Chiang Mai Hospital | Thailand | 2003-2011 | 2007 | 721 |  | 44,339 | Within 24 hours of anesthesia | 162.61 |  |  | Local anesthesia by surgeons, or underwent monitoring anesthesia |
| Yoon Ji Choi et al.  2014 | Asan Medical Center | Korea | 2004-2012 | 2009 | 19 |  | 18146 | Perioperative | 10.47 |  |  | Cardiac surgery or cardiac procedures |
| Young-Mu Kim et al. 2020 | Gangneung Asan Hospital | Korea | 2012-2018 | 2015 | 16 |  | 6588 | Within 24 hours of surgery | 24.29 |  |  | Cardiac arrest or CPCR before operating room |
| Huili Kan et al.  2021 | Liao Cheng People’s Hospital | China | 2013-2020 | 2017 | 25 |  | 19591 | Within 24 hours of anesthesia | 12.76 |  |  |  |
| **Death** | | | | | | | | | | | | |
| Charles J Vacanti et al. 1970 | Naval Hospitals | USA | 1964-1966 | 1965 |  | 126 | 10310 | Within 48 hours of operation |  | 122.21 |  |  |
| Jerite et al.  1973 | Bronx Municipal Hospital Center | USA | 1965-1969 | 1967 |  | 384 | 9931 | After 7 days of operation |  | 386.67 |  | Obstetric procedures |
| L. D. Karalliedde et al.1975 | General Hospital | Sri Lanka | 1972-1973 | 1973 |  | 6 | 2995 | Perioperative |  | 20.03 | 25 |  |
| S. C. Farrow et al.  1982 | The hospital in Cardiff | Welsh National | 1972-1977 | 1975 |  | 1038 | 19774 | hospital discharge |  | 524.93 |  |  |
| Richard L. Keenan et al.  1985 | Medical College of Virginia | USA | 1969-1983 | 1976 |  | 3 | 15300 | Within 9 days of operation |  | 1.96 | 70 |  |
| Bradley et al.  1998 | Princess Alexandra Hospital | Australia | 1983-1984 | 1983 |  | 64 | 2383 | Within 7 days of operation |  | 268.57 |  |  |
| AJ Heywood et al.1989 | the University Teaching Hospital | Zambia | 1987 | 1987 |  | 64 | 7857 | 6th postoperative day |  | 81.45 |  |  |
| T. Pedersen et al.  1989 | Herlev Hospital | Denmark | 1986-1987 | 1987 |  | 2 | 2454 | Within 24 hours of anesthesia |  | 8.15 |  |  |
| Ahmed et al.  2009 | Zaria | Nigeria | 1973-2000 | 1987 |  | 368 | 5201 | hospital discharge |  | 707.56 |  |  |
| Philippe Biboulet et al. 1998 | Lapeyronie University hospital | France | 1989-1995 | 1992 |  | 1 | 21676 | The first 12 postoperative  hours in the PACU or ICU |  | 0.46 | 50 | ASA 5 |
| Domingos Dias Cicarelli et al.  1998 | HC-FMUSP | Brazil | 1995 | 1995 |  | 114 | 6225 | Within 24 hours of anesthesia |  | 183.13 |  |  |
| A.A. Sanusi et al.  2001 | University College Hospital | Nigeria | 1994-1998 | 1996 |  | 20 | 1274 | Operating room |  | 156.99 | 16.67 |  |
| Roshan Ariyaratnam et.al.  2015 | Port Moresby General Hospital | Papua New Guinea | 1995-1999 | 1997 |  | 209 | 6044 | 30 days after operation |  | 345.80 |  |  |
| A. Ahmed et al.  2008 | Aga Khan University Hospital | Pakistan | 1992-2006 | 1999 |  | 15 | 24700 | Hospital discharge |  | 6.07 | 6.25 | Cardiac surgery |
| Roshan Ariyaratnam et.al.  2015 | Geelong Hospital | Australia | 2004-2012 | 2008 |  | 288 | 25562 | 30 days after operation |  | 112.67 |  |  |
| Yoon Ji Choi et al.  2014 | Asan Medical Center | Korea | 2004-2012 | 2009 |  | 15 | 18146 | Perioperative |  | 8.26 | 21.05 | Cardiac surgery or cardiac procedures |
| Roshan Ariyaratnam et.al.  2015 | National Minimum Datase | New Zealand | 2007-2011 | 2009 |  | 3718 | 306574 | 30 days after operation |  | 121.28 |  | Boarders, well babies, admissions solely for rehabilitation, or specific treatments such as dialysis and chemotherapy |
| Wangles Pignaton et al. 2016 | Hospital of the School of Medicine UNESP | Brazil | 2005-2012 | 2009 |  | 69 | 21371 | Perioperative period in the operation room and PACU |  | 32.29 |  |  |
| Rupert M Pearse et al. 2012 | 498 hospitals | 28 European nations | 2011 | 2011 |  | 732 | 11480 | 30 days after operation |  | 637.63 |  | <16yr of age or Patients undergoing planned day-case surgery, cardiac surgery, neurosurgery, radiological, or obstetric procedures |
| Jenny Lofgren et al. 2015 | Iganga General Hospital  Buluba Mission Hospital | Uganda | 2011 | 2011 |  | 15 | 1581 | 30 days after operation |  | 94.88 |  |  |
| Milagros Ruiz et al. 2015 | 11 hospitals | England | 2009-2012 | 2011 |  | 40749 | 885864 | 30 days after operation |  | 459.99 |  |  |
|  | 6 hospitals | Australia | 2009-2012 | 2011 |  | 14450 | 407807 | 30 days after operation |  | 354.33 |  |  |
|  | 5 hospitals | USA | 2009-2012 | 2011 |  | 11645 | 431698 | 30 days after operation |  | 269.75 |  |  |
|  | 6 hospitals | The Netherlands | 2009-2012 | 2011 |  | 6792 | 160086 | 30 days after operation |  | 424.27 |  |  |
| Perioperative Mortality Review Committee  2016 | Health Quality & Safety Commission | New Zealand | 2009-2013 | 2011 |  | 4976 | 293765 | 30 days after operation |  | 169.39 |  |  |
| Elizabeth L et al.  2015 | NACOR | USA | 2010-2014 | 2012 |  | 177 | 82324 | Within 48 hours of anesthesia |  | 21.50 |  | Analgesia for vaginal delivery |
| Jessica F. Davies et al. 2016 | 7 surgical hospitals Democratic | Republic of Congo Central African  Republic  South Sudan | 2011-2013 | 2012 |  | 769 | 7803 | 30 days after operation |  | 216.58 |  |  |
| Roshan Ariyaratnam et.al.  2015 | Greys Hospital | South Africa | 2010-2014 | 2013 |  | 156 | 3364 | 30 days after operation |  | 463.73 |  |  |
| Jennifer L. Rickard et al.  2016 | University  Teaching Hospital of Kigali | Rwanda | 2013 | 2013 |  | 157 | 1792 | 30 days after operation |  | 876.11 |  | obstetrics/gynecology, otorhinolaryngology and ophthalmology |
| Thomas J. Hopkins et al.  2016 | American Anesthesiology Inc | USA | 2009-2014 | 2013 |  | 358 | 28214 | Within 48 hours of procedure |  | 126.89 |  | ASA 6 |
| Mariko Sato et al.  2020 | Nara Medical University | Japan | 2008-2017 | 2013 |  | 37 | 7475 | Within 48 hours of anesthesia |  | 49.50 |  | Without anesthesiologists |
| B M Biccard et al.  2015 | 50 participating hospitals | South Africa | 2014 | 2014 |  | 98 | 2120 | 30 days after operation |  | 462.26 |  | cardiac surgery  <16yr of age |
| Young-Mu Kim et al. 2020 | Gangneung Asan Hospital | Korea | 2012-2018 | 2015 |  | 14 | 6588 | Within 24 hours of surgery |  | 21.25 | 12.5 | Cardiac arrest or CPCR before operating room |
| Danny et al.  2017 | 274 hospitals, UK Australia | New Zealand | 2020 | 2017 |  | 157 | 6617 | 30 days after operation |  | 273.27 |  | Cesarean sections,  ASA-PS |
