# Supplementary material for: Mortality and cardiac arrest rates of emergency surgery in developed and developing countries: a systematic review and meta-analysis
Source: BMC Anesthesiol. 2024 May 20;24:178. doi: 10.1186/s12871-024-02559-w (PMC11104000; doi:10.1186/s12871-024-02559-w)
Supplement: Supplementary file 2 — Supplementary Material 2 [file 12871_2024_2559_MOESM2_ESM.docx]

1. Vacanti CJ, VanHouten RJ, Hill RC: **A statistical analysis of the relationship of physical status to postoperative mortality in 68,388 cases**. *Anesth Analg* 1970, **49**(4):564-566.

2. Marx GF, Mateo CV, Orkin LR: **Computer analysis of postanesthetic deaths**. *Anesthesiology* 1973, **39**(1):54-58.

3. Farrow SC, Fowkes FG, Lunn JN, Robertson IB, Samuel P: **Epidemiology in anaesthesia. II: Factors affecting mortality in hospital**. *Br J Anaesth* 1982, **54**(8):811-817.

4. Keenan RL, Boyan CP: **Cardiac arrest due to anesthesia. A study of incidence and causes**. *JAMA* 1985, **253**(16):2373-2377.

5. P. P. Ruiz Neto, R. V. Gomide Amaral: **Parada Cardiaca durante a Anestesiaem um Complexo HospitalarEstudo Descritivo.** *Rev Bras Anest* 1986, **36**(2): 149-158.

6. Bradley JP, Beem MF, O'Donnell JE, van de Meene AH: **Mortality audit in a large teaching hospital**. *Anaesth Intensive Care* 1988, **16**(1):94-97.

7. Heywood AJ, Wilson IH, Sinclair JR: **Perioperative mortality in Zambia**. *Ann R Coll Surg Engl* 1989, **71**(6):354-358.

8. Pedersen T, Johansen SH: **Serious morbidity attributable to anaesthesia. Considerations for prevention**. *Anaesthesia* 1989, **44**(6):504-508.

9. Domingos Dias Cicarelli, Alessandra de Oliveira Mendes Gotardo: **Incidência de Obitos Anestésico-CirÃrgicos nas Primeiras 24Horas.Revisao de Prontuários de 1995 no Hospital dasClinicas da FMUSP**. *Rev Bras Anestesiol* 1998; **48**(4): 289-294

10. Biboulet P, Aubas P, Dubourdieu J, Rubenovitch J, Capdevila X, d'Athis F: **Fatal and non fatal cardiac arrests related to anesthesia**. *Can J Anaesth* 2001, **48**(4):326-332.

11. Sanusi AA, Soyannwo A, Amanor-Boadu SD: **Intra-operative cardiac arrests**. *West Afr J Med* 2001, **20**(4):192-195.

12. Aroonpruksakul N, Raksakiatisak M, Thapenthai Y, Wangtawesaup K, Chaiwat O, Vacharaksa K, Lertakyamanee J: **Perioperative cardiac arrest at Siriraj Hospital between 1999-2001**. *J Med Assoc Thai* 2002, **85 Suppl 3**:S993-999.

13. Braz LG, Braz JR, Modolo NS, Nascimento Junior P, Shuhama AP, Navarro LH: **[Cardiac arrest during anesthesia at a tertiary teaching hospital: prospective survey from 1996 to 2002.]**. *Rev Bras Anestesiol* 2004, **54**(6):755-768.

14. Braz LG, Modolo NS, do Nascimento P, Jr., Bruschi BA, Castiglia YM, Ganem EM, de Carvalho LR, Braz JR: **Perioperative cardiac arrest: a study of 53,718 anaesthetics over 9 yr from a Brazilian teaching hospital**. *Br J Anaesth* 2006, **96**(5):569-575.

15. Ahmed A, Ali M, Khan EA, Khan MU: **An audit of perioperative cardiac arrests in a Southeast Asian university teaching hospital over 15 years**. *Anaesth Intensive Care* 2008, **36**(5):710-716.

16. Ahmed A: **Trends in emergency surgical admissions in a tertiary health centre in Nigeria**. *West Afr J Med* 2009, **28**(2):106-109.

17. Goswami S, Brady JE, Jordan DA, Li G: **Intraoperative cardiac arrests in adults undergoing noncardiac surgery: incidence, risk factors, and survival outcome**. *Anesthesiology* 2012, **117**(5):1018-1026.

18. Pearse RM, Moreno RP, Bauer P, Pelosi P, Metnitz P, Spies C, Vallet B, Vincent JL, Hoeft A, Rhodes A *et al*: **Mortality after surgery in Europe: a 7 day cohort study**. *Lancet* 2012, **380**(9847):1059-1065.

19. Siriphuwanun V, Punjasawadwong Y, Lapisatepun W, Charuluxananan S, Uerpairojkit K: **Incidence of and factors associated with perioperative cardiac arrest within 24 hours of anesthesia for emergency surgery**. *Risk Manag Healthc Policy* 2014, **7**:155-162.

21 Yoon Ji Choi, Seon-uk Han, Seunghoon Woo, Young-Jin Ro, Hong-Seuk Yang: **Perioperative cardiac arrest in 457,529 anesthetized patients at a single teaching hospital in Korea: a retrospective study**. *Anesth Pain Med* 2014(9): 144-151

21. Biccard BM, Madiba TE, South African Surgical Outcomes Study I: **The South African Surgical Outcomes Study: A 7-day prospective observational cohort study**. *S Afr Med J* 2015, **105**(6):465-475.

22 Ariyaratnam R, Palmqvist CL, Hider P, Laing GL, Stupart D, Wilson L, Clarke DL, Hagander L, Watters DA, Gruen RL: **Toward a standard approach to measurement and reporting of perioperative mortality rate as a global indicator for surgery**. *Surgery* 2015, **158**(1):17-26.

23. Lofgren J, Kadobera D, Forsberg BC, Mulowooza J, Wladis A, Nordin P: **District-level surgery in Uganda: Indications, interventions and perioperative mortality**. *Surgery* 2015, **158**(1):7-16.

24. Ruiz M, Bottle A, Aylin PP: **The Global Comparators project: international comparison of 30-day in-hospital mortality by day of the week**. *BMJ Qual Saf* 2015, **24**(8):492-504.

25. Whitlock EL, Feiner JR, Chen LL: **Perioperative Mortality, 2010 to 2014: A Retrospective Cohort Study Using the National Anesthesia Clinical Outcomes Registry**. *Anesthesiology* 2015, **123**(6):1312-1321.

26. Davies JF, Lenglet A, van Wijhe M, Ariti C: **Perioperative mortality: Analysis of 3 years of operative data across 7 general surgical projects of Medecins Sans Frontieres in Democratic Republic of Congo, Central African Republic, and South Sudan**. *Surgery* 2016, **159**(5):1269-1278.

27. Hider P, Frampton C, Theis JC, Wilson L, Rothwell A: **Perioperative mortality in New Zealand related to hip and knee replacement surgery: comparing administrative and registry data**. *N Z Med J* 2016, **129**(1432):33-40.

28. Hopkins TJ, Raghunathan K, Barbeito A, Cooter M, Stafford-Smith M, Schroeder R, Grichnik K, Gilbert R, Aronson S: **Associations between ASA Physical Status and postoperative mortality at 48 h: a contemporary dataset analysis compared to a historical cohort**. *Perioper Med (Lond)* 2016, **5**:29.

29 Pignaton W, Braz JRC, Kusano PS, Modolo MP, de Carvalho LR, Braz MG, Braz LG: **Perioperative and Anesthesia-Related Mortality: An 8-Year Observational Survey From a Tertiary Teaching Hospital**. *Medicine (Baltimore)* 2016, **95**(2):e2208.

30. Kim YM, Lee JH, Kim HS, Kim JS, Yang HS: **Analysis of perioperative cardiac arrest in a rural hospital in Korea**. *Anesth Pain Med (Seoul)* 2020, **15**(3):325-333.

31. Sato M, Ida M, Naito Y, Kawaguchi M: **Perianesthetic death: a 10-year retrospective observational study in a Japanese university hospital**. *JA Clin Rep* 2020, **6**(1):8.

32. Wong DJN, Harris S, Sahni A, Bedford JR, Cortes L, Shawyer R, Wilson AM, Lindsay HA, Campbell D, Popham S *et al*: **Developing and validating subjective and objective risk-assessment measures for predicting mortality after major surgery: An international prospective cohort study**. *PLoS Med* 2020, **17**(10):e1003253.

33. Kan H, Ding Y, Wu S, Zhang Z: **Retrospective study of perioperative cardiac arrest from a Chinese tertiary hospital**. *Medicine (Baltimore)* 2021, **100**(32):e26890.
